# Effects of LAB Inoculants on the Fermentation Quality, Chemical Composition, and Bacterial Community of Oat Silage on the Qinghai-Tibetan Plateau

**DOI:** 10.3390/microorganisms10040787

**Published:** 2022-04-08

**Authors:** Qiming Cheng, Liangyin Chen, Yulian Chen, Ping Li, Chao Chen

**Affiliations:** 1 College of Animal Science, Guizhou University, Guiyang 550025, China; qmcheng@gzu.edu.cn (Q.C.); lychen2011430@sina.com (L.C.); ylchen1@gzu.edu.cn (Y.C.); 2 Key Laboratory of Animal Genetics, Breeding and Reproduction in the Plateau Mountainous Region, Ministry of Education, Guizhou University, Guiyang 550025, China; 3 Sichuan Academy of Grassland Sciences, Chengdu 611731, China

**Keywords:** lactic acid bacteria, phenyllactic acid, fermentation, bacterial community, silage

## Abstract

Lactic acid bacteria (LAB) have been proposed for the control of undesirable fermentation and, subsequently, aerobic deterioration due to their ability to produce antimicrobial metabolites in silage mass. To investigate the effect of specific LAB on the silage fermentation characteristics and bacterial community composition of oat in cold regions, silages were treated without (control) or with three LAB strains (LB, *Lentilactobacillus buchneri*; nLP, low temperature tolerant *Lactiplantibacillus plantarum*; pLP, phenyllactic acid-producing *Lactiplantibacillus plantarum*), and then stored at ambient temperature (−2.63 ± 5.47–14.29 ± 5.48 °C) for 30, 60, and 90 days. Compared with control, inoculation of LAB decreased the final pH value, butyric acid content, ammonia-N of total N and dry matter loss of silage. Treatments with nLP and pLP increased (*p* < 0.05) lactic acid content, whereas LB increased (*p* < 0.05) acetic acid content of silage. *Lactiplantibacillus* and *Leuconostoc* dominated in the silages with relative abundance of 68.29–96.63%. A prolonged storage period enhanced the growth of *Leuconostoc* in pLP-treated silage. In addition, pLP increased (*p* < 0.05) the aerobic stability of silage as compared with nLP. In conclusion, inoculation of LAB improved silage fermentation and/or delayed aerobic deterioration by shifting bacterial community composition during ensiling. Phenyllactic acid-producing *Lactiplantibacillus* *plantarum* as an inoculant exhibited potential for high quality silage production.

## 1. Introduction

The Qinghai-Tibetan Plateau is a key alpine grassland livestock production area [1]. However, in the winter and early spring, the inclement weather causes food shortages for herbivores, resulting in yak weight loss, decreased milk production, and other issues [2,3]. Therefore, how to effectively preserve local forages is an increasing issue in cold regions.

Ensiling is an important technique for preserving fresh forage, especially in areas where forage is seasonally or regionally unbalanced due to the harsh conditions. In cold regions, silage is considered to be the main feed source for ruminants [4]. However, the heavy distribution of undesirable microorganisms such as aerobic bacteria, yeasts, and molds during ensiling and after exposure to air is a concerning issue in the world. Microbial additives have been applied to specifically elevate dominance of bacterial genera such as *Lactiplantibacillus*, *Pediococcus,* and *Leuconostoc* for optimizing the silage fermentation process. Lactic acid bacteria (LAB) have been used as microbial additives because they produce metabolites that inhibit harmful microorganisms during silage fermentation, including organic acids, fatty acids, ethonal hydrogen peroxide, acetoin, diacetyl, cyclic dipeptides, bacteriocins, or bacteriocin-like inhibitory sub-stances [5]. However, lower ambient temperatures can undesirably delay or stop production of lactic and acetic acids during ensiling in cold regions [6]. This is because the activity of lactic acid bacteria is lower in silage under low temperatures, while the activity of yeast and other harmful microorganisms is higher [7,8]. Recently, some researchers have begun to focus on the potential of low temperature tolerant LAB strains for ensiling [9,10]. Previously published studies indicated that species of *Lentilactobacillus buchneri* and/or low temperature tolerant *Lactiplantibacillus plantarum* is combined in microbial inoculants for functionally advancing *Phalaris arundinacea* silage fermentation process and reducing aerobic spoilage initiated by lactate-assimilating yeasts in cold region [11]. However, there is little information on the effect of a low temperature tolerant LAB inoculant on the fermentation characteristics, bacterial community, and aerobic stability of oat silage in a cold region.

Phenyllactic acid, as an eco-friendly antimicrobial agent with high stability under low temperature conditions [12], can inhibit the growth of pathogenic bacteria, yeasts, and molds in foods [13]. Phenyllactic acid is also being investigated for animal feeding and disease prevention as an alternative to the use of antibiotic substances [14]. Some species of LAB, such as *Lactiplantibacillus plantarum*, *Lacticaseibacillus casei,* and *Pediococcus acidilactici,* can produce phenyllactic acid [15]. Ström et al. (2002) [16] first isolated a *Lactiplantibacillus plantarum* strain (MiLAB 393) from grass silage that produces broad-spectrum antifungal compounds, active against food- and feed-borne filamentous fungi and yeasts in a dual-culture agar plate assay. In our laboratory, a LAB strain, *Lactiplantibacillus plantarum,* was isolated from natural fermented-oat silage on the Qinghai Tibetan Plateau and exhibited a high phenyllactic acid producing ability of 94.2 mg/mL in de Man, Rogosa and Sharpe (MRS) broth incubated at 28 °C for 24 h (data not published). However, limited information is available on how the phenyllactic acid-producing LAB strain affects fermentation quality and how phenyllactic acid regulates the microbiota of silage.

In addition, amounts of fresh oats were left on the land due to unstable weather conditions on the Qinghai Tibetan Plateau. The ambient temperature falls to <15 °C, which usually results in unfinished fermentation during ensiling and rapid deterioration after exposure to air [17]. Hence, the objective of the present study was to compare the effects of specific LAB, including *Lentilactobacillus buchneri*, low temperature tolerant *Lactiplantibacillus plantarum,* and phenyllactic acid-producing *Lactiplantibacillus plantarum,* on the fermentation characteristics, bacterial community composition, and aerobic stability of oat silage stored on the Qinghai Tibetan Plateau. Our hypothesis was that the three LAB strains could functionally improve the silage quality of oats under low storage temperature (<15 °C) conditions.

## 2. Materials and Methods

### 2.1. Silage Preparation

Experiment was conducted at the Hongyuan experimental base of Sichuan Academy of Grassland Sciences (44°53′ N, 7°41′ E, altitude 3500 m), which locates on the Qinghai Tibetan Plateau in P. R. China. Oat at milk stage was harvested as ensiling material at 10 September 2020.

The harvested oat was chopped to 1–3 cm by a chopper, and randomly divided into thirty-six 10-kg piles to obtain nine replications per treatment. The treatments were as follows: control without additives (CK); LB, *Lentilactobacillus buchneri* isolated from natural-fermented silage; nLP, a low temperature tolerant *Lactiplantibacillus plantarum* isolated from natural fermented-reed canary grass silage, can grow well at 5–30 °C [11,18]; pLPphenyllactic acid-producing *Lactiplantibacillus plantarum* isolated from natural fermented-oat silage and preserved at China General Microbiological Cultural Collection Center (No. 14117). To reduce negative effects from addition amounts, each LAB was applied at a rate of 10^6^ cfu/g of fresh matter (FM). The inoculum level of LAB was determined according to Gallo et al. (2021) [19]. Each LAB was separately diluted in sterilized water and sprayed uniformly onto the forage using a hand sprayer, which was constantly hand mixed and yield applying amount of 4 L inoculant-diluted solution/t of fresh forage. The same amount of water was added to the CK treatment. The treated forage from each pile was packed in a 20 L plastic silo equipped with a lid that only enabled gas release. The density of all silages was about 500 ± 25 kg/m^3^ on FM basis. Three of silage silos with same treatment were sampled after 30, 60, and 90 days of ensiling at ambient temperature (−2.63 ± 5.47–14.29 ± 5.48 °C). Samples from the fresh forage and the silages were subject to analysis of chemical composition, aerobic stability, microbial population, and/or bacterial community.

### 2.2. Chemical Analysis

Samples were dried at 65 °C for a constant weight to determine dry matter (DM) content, and then ground through 0.20 mm sieve for water soluble carbohydrates (WSC) analysis by the method of McDonald [20]. The DM loss was calculated by formula as follows: DM loss (%) = 100 × [1 − (pre-ensiled forage weight/silage weight at opening)].

Fresh sample of 20 g was mixed with 180 mL ultrapure water for 3 min in a stomacher blender. The pH of filtrate was determined by pH meter. Filtrate of about 10 mL was subjected to centrifugation (4500× *g*, 15 min, 4 °C), and the supernatant was analyzed for lactic acid, acetic acid, propionic acid, and butyric acid using high performance liquid chromatography [21]. Identification and quantification of phenyllactic acid were determined by the method of Jung et al. (2019) [13]. Ammonia nitrogen was determined by methods of Broderick and Kang [22].

### 2.3. Microbial Population Analysis

Microbial population on fresh samples was determined by the method of Cai [23]. Ten grams of each fresh sample were put into a sterile glass bottle, suspended in 90 mL of sterile water, and homogenized for 2 h in a laboratory blender (LB20ES, Shanghai Prime Science Co., Ltd., Shanghai, China). Serial dilutions were made. The number of LAB were counted on MRS agar (GCM188, Land Bridge Technology Co., Ltd., Beijing, China), incubated at 37 °C for 48 h. Yeasts were counted on malt extract agar with 1.5 mg/L Tetracycline (CM173, Land Bridge Technology Co., Ltd., Beijing, China), incubated at 30 °C for 48 h. Yeasts were distinguished from molds by colony appearance and observation of cell morphology.

### 2.4. Bacterial Community Analysis

The extraction of bacterial DNA from fresh sample was determined by the method of Li [21]. In brief, Phusion^®^ High-Fidelity polymerase chain reaction (PCR) Master Mix (New England Biolabs) was used to carry out PCR reactions, following the manufacturer’s instructions. The primers 515 F and 907 R was chosen to amplify the V4–V5 region of 16S rRNA gene. The PCR amplicons were then sequenced by using an Illumina MiSeq PE2500 platform at Novogene Company (Beijing, China). After sequencing, paired reads were merged using FLASH (V 1.2.7) and filtered by QIIME. The UPARSE method was employed to assign operational taxonomic units (OTUs) to the 16S rRNA at a cutoff level of 3% on the Usearch software platform (Version 7.1). Based on OTUs results, the alpha indices were calculated with QIIME (Version 1.7.0) and displayed with R software (Version 2.15.3).

### 2.5. Aerobic Stability

The aerobic stability of silage was measured by the method of Kung [24]. At silo opening, approximately 5.0 kg of silage from each silo was returned to clean buckets without packing under air-controlled temperature of 25 ± 0.5 °C. A thermocouple probe was placed in the geometric center of each silage mass, and temperatures were recorded by a data logger (YA204R, YADU Electronic Technology Co., Ltd., Shanghai, China) every 30 min. Silos were covered with 2 layers of cheesecloth and exposed to air. Aerobic stability was determined as the number of hours before the temperature of the silage mass increased 2 °C above ambient temperature of each silage mass.

### 2.6. Statistical Analysis

Data was analyzed as a 4 × 3 factorial arrangement in a completely randomized design. The model included the fixed effects of additive, storage period, and their interaction. Data were analyzed using the Fit Model procedure of JMP (SAS Institute Inc., Cary, NC, USA), and differences are reported as significant when *p* ≤ 0.05. Means were separated by Tukey’s test (*p* ≤ 0.05).

## 3. Results and Discussion

The chemical compositions and numbers of LAB and yeasts of silages were shown in Table 1. The additive, storage period, and their interaction significantly (*p* < 0.05) affected the DM loss and WSC of silages. The content WSC of silages tended to decrease, while DM loss tended to increase with storage period (*p* < 0.05). Moisture content of fresh forage is an important factor for silage fermentation. The suitable moisture content of fresh forage helps to effectively compact, achieve an anaerobic environment, and prevent mold or yeast growth [25]. A previous study has found that pre-ensiling forage with >35% DM may result in molding and heating clearly associated with difficulty in removing oxygen [26]. However, forage ensiled at <30% DM may increase leakage losses and promote clostridial deterioration, thereby reducing voluntary intake [27]. In our study, fresh forage with a water content exceeding 75% (DM, 24.55%) was not easily compacted, resulting in a packing density (500 ± 25 kg/m^3^) that was less than the recommended density of 600 kg/m^3^. As a result, oat silage without any treatment showed poor fermentation with pH of 4.45–4.55, butyric acid of 0.13–0.16%DM and ammonia-N of 14.3–18.72%TN (Table 2). 

**Figure 1:**
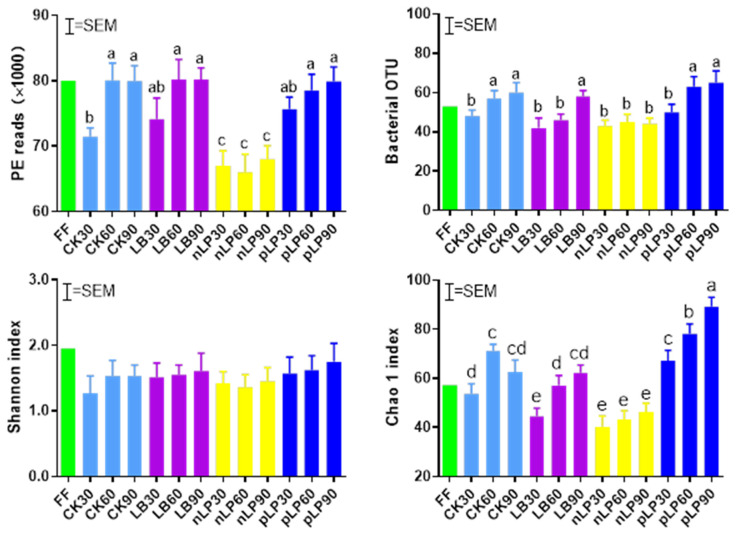
The bacterial community index of fresh forage (FF) and control without additives (CK) or with functional LAB (LB, *Lentilactobacillus buchneri*; nLP, low temperature tolerant *Lactiplantibacillus plantarum*; pLP, phenyllactic acid-producing *Lactiplantibacillus plantarum*; each application rate of 10^6^ cfu/g FM), and ensiled for 30 d, 60 d, and 90 d. Bars with different letters (a–e) differ (*p* < 0.05). OTU, operational taxonomic unit; SEM, standard error of means.

**Figure 2:**
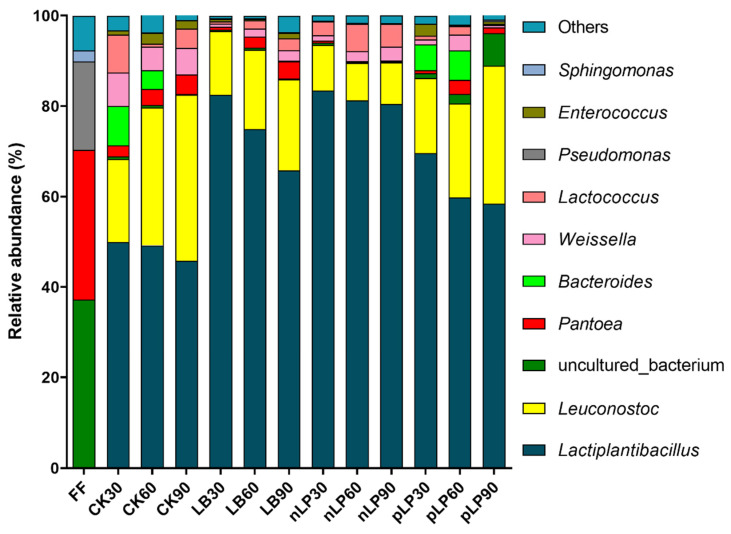
The relative abundance of top 10 bacterial genera of fresh forage (FF) and control without additives (CK) or with functional LAB (LB, *Lentilactobacillus buchneri*; nLP, low temperature tolerant *Lactiplantibacillus plantarum*; pLP, phenyllactic acid-producing *Lactiplantibacillus plantarum*; each application rate of 10^6^ cfu/g FM), and ensiled for 30 d, 60 d and 90 d.

The WSC content (8.91%DM) of fresh forage was sufficient for an adequate fermentation process during ensiling [28]. Inoculations of functional LAB treatments decreased DM loss as compared with the control silage in our study, because LAB causes a reduction in DM loss in silage fermentation [29]. The significantly higher DM loss reported in LB silage than in nLP and pLP silages was due to the production of carbon dioxide by LB (*Lentilactobacillus buchneri*) through heterofermentative fermentation, resulting in considerable DM losses [10]. However, the epiphytic LAB count (2.81 log cfu/g of FM) on the plants was below the minimum requirement (5.0 log cfu/g FM) for high quality silage [13]. In addition, the high yeast count (4.71 log cfu/g of FM) distributed on the plants may increase the potential for more DM loss during ensiling. The counts of LAB and yeasts of fresh forage in our study were lower than the results of Wang et al. (2020) [30], who reported that the LAB and yeast counts of fresh oat were 5.61 and 8.45 log cfu/g of FM, respectively. A similar situation was also observed by Chen et al. (2020) [18], who reported that both forages growing at different geographic locations but with similar maturity stages had inconsistent microbial composition and structure on the plants, which were caused by ambient temperature differences [31]. The additive significantly (*p* < 0.05) affected the LAB and yeast counts of silages. In our study, inoculations of functional LAB treatments promoted the growth of LAB and inhibited the growth of yeasts in silage, and the LAB counts of LB and nLP treatments were significantly (*p* < 0.05) higher than those of other treatments. This result was verified in our microbial diversity analysis data (Figure 1 and Figure 2).

The additive, storage period, and their interaction significantly (*p* < 0.05) affected the lactic acid, ammonia-N, and aerobic stability of silages, and the additive significantly affected pH and butyric acid (Table 2). One of the most essential markers for determining silage quality is the pH value. The growth of acid-intolerant and hazardous microbes such as *Clostridium* will be aided by a high pH value, resulting in poor silage fermentation. The growth of LAB will be inhibited, and the fermentation quality will be harmed if the pH is too low. Inoculations of functional LAB treatments lowered the pH value in silage in this investigation. Similar findings were reported by Li et al. (2019) [21], who found that the inoculation of exogenous LAB (*Lactiplantibacillus plantarum* and *Lentilactobacillus buchneri*) improved the fermentation quality of silage. It will promote LAB to produce lactic acid to reduce the pH value in silage (Table 2). Ammonia-N is also an important index for evaluating the quality of silage, which reflects the activity of plant proteases or the degree of protein degradation based on clostridial fermentation [32]. In our study, inoculations of functional LAB decreased ammonia-N of total N of silages, which indicated that functional LAB inhibited the growth and propagation of harmful microorganisms in silage, thereby reducing the degradation of protein. This result was verified in our microbial diversity analysis data (Figure 2 and Table 1). Another presentation of this result is that the butyric acid content of functional LAB treatments was significantly (*p* < 0.05) lower than that of the control. The fermentation quality of nLP silage was the best, mainly reflected in that nLP silage had the lowest pH (<4.2) and ammonia-N (10.25–12.13%TN), and the highest LA content (>3%DM). This indicated that the nLP strain could better play its role in low temperature environments than other LAB strains [3]. Inoculation of LB increased the acetic acid content of silage. A similar result on oat silage was from Gomes et al., 2019 [33]. Moreover, the concentration of lactic acid occurred at its highest and changed little in the nLP-inoculated silage. This was probably because of the metabolism of the exogenous strains of *Lactiplantibacillus plantarum*, which is functionally capable of quickly producing lactic acid under low temperature conditions [18].

In the present study, LB (*Lentilactobacillus buchneri*) silage had the highest (*p* < 0.05) aerobic stability (>170 h) of the other silages. The use of *Lentilactobacillus buchneri* to improve aerobic stability has proved valuable due to its production of acetic acid [24]. A similar situation was found by Kleinschmit and Kung [34], who evaluated 43 studies that inoculated *Lentilactobacillus buchneri* in different forages to ensilage and reported that the inoculant improved the aerobic stability of silages. In fact, the aerobic stability of pLP inoculated silages also exhibited higher stability than control and nLP inoculated silages (Table 2). This may be due to the fact that production of phenyllactic acid (14.4–16.8‰DM) and acetic acid (0.36–0.38%DM) during ensiling can penetrate into the cell membranes of microorganisms, destroying their biological activity and inhibiting their growth [13,35]. Previous studies found that a negative relationship between aerobic stability and the number of yeasts [36]. In the present study, the yeast count of pLP silage was higher than nLP silage, and the aerobic stability was also higher than pLP treatments. The likely reason for this result is that counting yeasts on malt extract agar could not distinguish between yeasts that are capable of assimilating lactic acid compared with those that are not, and it is these former species that cause aerobic spoilage in many silages [24]. The aerobic stability gradually increased as the storage period prolonged, which was consistent to the results of Kung et al. (2018) [24], who reported that the ensiling time prolonged can increase the aerobic stability of corn silage.

Phenyllactic acid has recently been proposed for its activities against bacteria, yeasts, and molds in foods [13] and animal diets [14] for their very low toxicity for animals and humans. In addition, phenyllactic acid could be produced at a low cost by using effective fermentation modes through LAB [37]. In our study, a high phenyllactic acid content (14.4–16.8‰DM) was detected in pLP silage but not in the other silages. This indicated that the phenyllactic acid produced by the pLP strain could be effectively used as an antifungal compound to delay the growth of a variety of fungal contaminants and to also extend the shelf life of feed stuffs [38]. At the same time, phenyllactic acid-producing LAB exhibited potential for enhancing the quality of fermented products. Compared with LB and control, the inoculation of pLP did shift fermentation characteristics with high lactic acid (2.43–2.86%DM) and low butyric acid (0.05–0.06%DM) of silage. However, the pLP inoculated silage showed higher (*p* < 0.05) ammonia-N of total N and numbers of yeasts and lower LAB than the nLP inoculated silage. This may be due to the fact that low-temperature tolerant *Lactiplantibacillus plantarum* could be robust for rapid pH reduction to inhibit plant inherent proteolysis and undesirable microorganisms such as *Clostridia* [18]. Another possible reason is that phenyllactic acid could inhibit the growth of LAB [39].

It is well known that the natural fermentation of forages depends on epiphytic microflora, especially the count of LAB in an anaerobic environment [40]. In addition, various bacterial communities and successions have been found in different pre- and post-silage forages [41]. Therefore, bacterial community composition plays a vital role in silage fermentation and knowing community composition is a necessary condition to understand the complex process of ensiling [42]. Next generation sequencing could help us better understand the silage fermentation pattern. In the present study, the bacterial alpha diversity indices of OTU and Chao 1 increased as the storage period prolonged (Figure 1). This may be due to the higher silage pH of >4.2, which exerts a limited effect on most undesirable acid-tolerant microbes in silage [21]. nLP-treated silage had lower PE reads, OTU and Chao1 than other treatments, which suggested that low temperature tolerant LAB inoculant could be robust for rapid pH reduction to inhibit the growth of other bacteria [18]. However, silage treated with pLP showed a higher diversity as compared with control. This may be because phenyllactic acid inhibited the growth of LAB, thereby promoting the growth of other microorganisms. The main microorganisms of fresh oat were uncultured bacterium (37.13%), *Pantoea* (33.12%), and *Pseudomonas* (19.55%), which differs from previous results showing that *Enterobacter*, *Pantoea,* and *Serraia* were the predominant genera in fresh soybeans [43]; additionally, *Agrobacterium*, *Microbacterium,* and *Sphingobacterium* were dominated in the microbial composition in fresh whole crop corn [42]. Previous studies have illustrated that the colonization of plant surfaces by bacteria depends on many factors, including material species, climate, period of duration, geographical location, solar radiation intensity, and the type of fertilizer used [20,22,44].

Over the 30–90 d of ensiling, the bacterial communities in the silages were highly dominated by the genera belonging to LAB, while undesirable microbial communities were extensively inhibited (Figure 2). The genera of *Lactiplantibacillus* and *Leuconostoc* dominated the silages, with a relative abundance of 68.29–96.63%. A similar observation was reported by Xu et al. (2019) [42], who found that most of the undesirable microorganisms were inhibited after fermentation, while *Lactiplantibacillus* (>98%) was the dominant genera in corn silages stored for 90 days. *Lactiplantibacillus* is a rod-shaped LAB that can convert plant carbohydrates into LA to decline pH value of silage. In our study, nLP silages had a higher relative abundance of *Lactiplantibacillus* than the other silages. This is the main reason why nLP silages had the highest LA content and the lowest pH than other group silages (Table 2). This proves once again that low temperature-tolerant LAB inoculan could better play its role in cold regions than other LAB strains [18]. The nLP-inoculated silage showed a low relative abundance of *Leuconostoc* and a high relative abundance of *Lactiplantibacillus* relative to control, LB, and nLP silages. This is the main reason why there is lower (*p* < 0.05) acetic acid content and higher (*p* < 0.05) lactic acid content in nLP silage than in other silages. *Leuconostoc* performs heterolactic acid fermentation and can metabolize diverse organic compounds to produce acetic acid [45]. Compared with control, inoculations of functional LAB (LB, nLP, and pLP) increased the relative abundance of *Lactiplantibacillus* by 21.62–75.88% but decreased that of *Leuconotoc* by 16.88–75.05% in silages. A prolonged storage period reduced the positive effects from LAB inoculation on the increase in relative abundance of *Lactiplantibacillus* and the decrease in relative abundance of *Leuconostoc* in silage, which increased the potential for aerobic deterioration [46]. The role of *Pantoea* species in silage fermentation is unclear. Previous studies have found that *Pantoea* can reduce the content of NH_3_-N [47]. In contrast, Li et al. (2017) [48] thought *Pantoea* in silage had a similar effect to *Enterobacter* in that they compete for nutrients with LAB, implying that *Pantoea* in silage would be undesirable as well. In our study, the relative abundance of *Pantoea* was decreased significantly after silage fermentation, and the content in nLP-treated silage was the lowest.

## 4. Conclusions

This study showed that inoculation of specific LAB at ensiling could improve oat silage quality by reconstructing bacterial community composition. Both *Lentilactobacillus buchneri* and pheneyllactic acid-produced *Lactiplantibacillus plantarum* increased the aerobic stability of silage. In particular, low temperature-tolerant *Lactiplantibacillus plantarum* inoculan could better play its role in cold regions than other LAB strains.

## Figures and Tables

**Table 1:** The chemical and microbial compositions of fresh forage and silages after 30, 60, and 90 days of ensiling.

Items	Fresh Forage	Additives	Storage Period			SEM	*p*-Value		
			30 d	60 d	90 d		A	S	A × S
DM, %	24.55	CK	25.46	26.59	25.72	0.72	0.197	0.241	0.508
		LB	26.12	27.75	25.27				
		nLP	25.29	26.18	26.44				
		pLP	26.38	26.00	26.82				
DM loss, %	_	CK	3.17^Ac^	3.48^Ab^	3.86^Aa^	0.26	<0.001	<0.001	<0.001
		LB	2.65^Bb^	2.82^Bb^	3.41^Ba^				
		nLP	1.81^Db^	1.96D^ab^	2.08^Da^				
		pLP	2.21^Cb^	2.34C^ab^	2.58^Ca^				
WSC, %DM	8.91	CK	2.18^Ba^	1.98^Ba^	0.17^Bb^	1.02	<0.001	<0.001	<0.001
		LB	2.15^Ba^	1.87^Bb^	0.20^Bc^				
		nLP	3.19^Aa^	2.28^Ab^	2.16^Ab^				
		pLP	3.21^Aa^	2.19^Ab^	0.32^Bc^				
LAB, log cfu/g of FM	2.81	CK	5.55^D^	5.43^C^	5.58^C^	0.54	<0.001	0.137	<0.001
		LB	8.12^Bb^	9.42^Aa^	9.66^Aa^				
		nLP	9.45^A^	9.88^A^	9.52^A^				
		pLP	6.89^C^	7.45^B^	7.16^B^				
Yeasts, log cfu/g of FM	4.17	CK	3.1	3.36	3.48	—	—	—	—
		LB	<1.0	<1.0	<1.0				
		nLP	<1.0	<1.0	<1.0				
		pLP	2.22	2.14	2.1				

A, additive; A × S, interaction between additive and storage period; CK, control without additives; DM, dry matter; FM, fresh matter; LAB, lactic acid bacteria; LB, *Lentilactobacillus buchneri*; nLP, low temperature tolerant *Lactiplantibacillus plantarum*; pLP, phenyllactic acid-producing *Lactiplantibacillus plantarum*; S, storage period; SEM, standard error of mean; WSC, water soluble carbohydrates. Values with different letters in the same column are significantly different (*p* < 0.05).

**Table 2:** The fermentation quality and aerobic stability of silages after 30, 60, and 90 days of ensiling.

Items	Additives	Storage Period			SEM	*p*-Value		
		30 d	60 d	90 d		A	S	A × S
pH	CK	4.55	4.45	4.51	0.06	<0.001	0.056	0.115
	LB	4.41	4.34	4.38				
	nLP	4.18	4.19	4.14				
	pLP	4.42	4.41	4.44				
Phenyllactic acid, %DM	CK	<0.1	nd	nd	—	—	—	—
	LB	nd	nd	nd				
	nLP	nd	nd	nd				
	pLP	16.8	14.4	15.1				
Lactic acid, % DM	CK	2.02^D^	1.88^C^	1.89^C^	0.39	<0.001	<0.001	<0.001
	LB	2.26^C^	2.22^B^	2.23^B^				
	nLP	3.02^A^	3.19^A^	3.06^A^				
	pLP	2.86^Ba^	2.49^Bb^	2.43^Bb^				
Acetic acid, % DM	CK	0.24	0.28	0.32	0.14	0.008	0.195	0.466
	LB	0.71	0.79	0.8				
	nLP	0.17	0.18	0.16				
	pLP	0.36	0.38	0.37				
Propionic acid, % DM	CK	nd	nd	0.14	0.01	—	—	—
	LB	0.25	0.2	0.22				
	nLP	nd	nd	0.05				
	pLP	nd	0.15	0.17				
Butyric acid, % DM	CK	0.16	0.14	0.13	0.01	<0.001	0.216	0.179
	LB	0.1	0.08	0.09				
	nLP	0.08	0.09	0.09				
	pLP	0.06	0.06	0.05				
Ammonia-N, %TN	CK	14.30^Ab^	18.72^Aa^	18.50^Aa^	1.04	<0.001	<0.001	<0.001
	LB	12.61^Bc^	14.27^Bb^	15.07^Ba^				
	nLP	10.25^Cb^	11.48^Cab^	12.13^Ca^				
	pLP	12.31^Bc^	13.42^Bb^	14.97^Ba^				
Aerobic stability, h	CK	86.00^Db^	84.00^Db^	98.00^Ca^	8.41	<0.001	<0.001	<0.001
	LB	171.00^A^	179.00^A^	184.00^A^				
	nLP	96.00^C^	100.00^C^	98.00^C^				
	pLP	118.00^Bb^	122.00^Bb^	142.00^Ba^				

A, additive; A × S, interaction between additive and storage period; CK, control without additives; DM, dry matter; LB, *Lentilactobacillus buchneri*; nd, no detected; nLP, low temperature tolerant *Lactiplantibacillus plantarum*; pLP, phenyllactic acid-producing *Lactiplantibacillus plantarum*; S, storage period; SEM, standard error of mean; TN, total nitrogen. Values with different letters in the same column are significantly different (*p* < 0.05).

## Data Availability

Data is contained within the article.

## References

[1] Guo P., Gao P., Li F., Chang S., Wang Z., Yan T. (2020). Prediction of Metabolizable Energy Concentrations of Herbage in the Qinghai–Tibetan Plateau Using Tibetan Sheep Digestibility Data. Animals.

[2] Zhou J., Liu H., Zhong C. (2018). Apparent Digestibility, Rumen Fermentation, Digestive Enzymes and Urinary Purine Derivatives in Yaks and Qaidam Cattle Offered Forage-Concentrate Diets with Different Nitrogen Concentration. Livest. Sci..

[3] Long R.J., Zhang D.G., Wang X., Hu Z.Z., Dong S.K. (1999). Effect of Strategic Feed Supplementation on Productive and Reproductive Performance in Yak Cows. Prev. Veter. Med..

[4] Wilkinson J.M., Rinne M. (2018). Highlights of Progress in Silage Conservation and Future Perspectives. Grass Forage Sci..

[5] Crowley S., Mahony J., van Sinderen D. (2013). Current Perspectives on Antifungal Lactic Acid Bacteria as Natural Bio-Preservatives. Trends Food Sci. Technol..

[6] Bernardes T.F., Daniel J., Adesogan A.T., McAllister T.A., Drouin P., Nussio L.G., Huhtanen P., Tremblay G.F., Bélanger G., Cai Y. (2018). Silage Review: Unique Challenges of Silages Made in Hot and Cold Regions. J. Dairy Sci..

[7] Kung L., Shaver R.D., Gran R.J. (2018). Silage Review: Interpretation of Chemical, Microbial and Organoleptic Components of Silages. J. Dairy Sci..

[8] Gourama H., Bullerman L.B. (1995). Inhibition of Growth and Aflatoxin Production of Aspergillus Flavus by *Lactobacillus* Species. J. Food Protect..

[9] Ali G., Liu Q., Yuan X., Dong Z., Desta S.T., Li J., Bai X., Shah A.A., Shao T. (2017). Characteristics of Lactic Acid Bacteria Isolates and Their Effects on the Fermentation Quality of Acacia (*Sophora Japonica* L.) Leaf Silage at Low Temperatures. Grasl. Sci..

[10] Zhang M., Lv H., Tan Z., Li Y., Wang Y., Pang H., Li Z., Jiao Z., Jin Q. (2017). Improving the Fermentation Quality of Wheat Straw Silage Stored at Low Temperature by Psychrotrophic Lactic Acid Bacteria. Anim. Sci. J..

[11] Chen L., Li P., Gou W., You M., Cheng Q., Bai S., Cai Y. (2020). Effects of Inoculants on the Fermentation Characteristics and in Vitro Digestibility of Reed Canary Grass (*Phalaris Arundinacea* L.) Silage on the Qinghai-Tibetan Plateau. Anim. Sci. J..

[12] Cortés-Zavaleta O., López-Malo A., Hernandez-Mendoza A., García H.S. (2014). Antifungal Activity of *Lactobacilli* and Its Relationship with 3-Phenyllactic Acid Production. Int. J. Food Microbiol..

[13] Jung S., Hwang H., Lee J.-H. (2019). Effect of Lactic Acid Bacteria on Phenyllactic Acid Production in Kimchi. Food Control.

[14] Kim D.W., Kim J.H., Kang H.K., Akter N., Kim M.J., Na J.C., Hwangbo J., You S.W., Choi H.C., Suh O.S. (2014). Dietary Supplementation of Phenyllactic Acid on Growth Performance, Immune Response, Cecal Microbial Population, and Meat Quality Attributes of Broiler Chickens. J. Appl. Poult. Res..

[15] Bustos A.Y., de Valdez G.F., Gerez C.L. (2018). Optimization of Phenyllactic Acid Production by *Pediococcus Acidilactici* CRL 1753. Application of the Formulated Bio-Preserver Culture in Bread. Biol. Control.

[16] Ström K., Sjögren J., Broberg A., Schnürer J. (2002). *Lactobacillus Plantarum* MiLAB 393 Produces the Antifungal Cyclic Dipeptides Cyclo (l-Phe-l-Pro) and Cyclo (l-Phe-trans-4-OH-l-Pro) and 3-Phenyllactic Acid. Appl. Environ. Microbiol..

[17] Wang C., Nishino N. (2013). Effects of Storage Temperature and Ensiling Period on Fermentation Products, Aerobic Stability and Microbial Communities of Total Mixed Ration Silage. J. Appl. Microbiol..

[18] Chen L., Bai S., You M., Xiao B., Li P., Cai Y. (2020). Effect of a Low Temperature Tolerant Lactic Acid Bacteria Inoculant on the Fermentation Quality and Bacterial Community of Oat Round Bale Silage. Anim. Feed Sci. Technol..

[19] Gallo A., Fancello F., Ghilardelli F., Zara S., Froldi F., Spanghero M. (2021). Effects of Several Lactic Acid Bacteria Inoculants on Fermentation and Mycotoxins in Corn Silage. Anim. Feed Sci. Technol..

[20] McDonald P., Henderson N., Heron S. (1991). The Biochemistry of Silage.

[21] Li P., Zhang Y., Gou W., Cheng Q., Bai S., Cai Y. (2019). Silage Fermentation and Bacterial Community of Bur Clover, Annual Ryegrass and Their Mixtures Prepared with Microbial Inoculant and Chemical Additive. Anim. Feed Sci. Technol..

[22] Broderick G.A., Kang J.H. (1980). Automated Simultaneous Determination of Ammonia and Total Amino Acids in Ruminal Fluid and In Vitro Media. J. Dairy Sci..

[23] Cai Y., Benno Y., Ogawa M., Kumai S. (1999). Effect of Applying Lactic Acid Bacteria Isolated from Forage Crops on Fermentation Characteristics and Aerobic Deterioration of Silage. J. Dairy Sci..

[24] Kung L., Smith M.L., Da Silva E.B., Windle M.C., Da Silva T.C., Polukis S.A. (2018). An Evaluation of the Effectiveness of a Chemical Additive Based on Sodium Benzoate, Potassium Sorbate, and Sodium Nitrite on the Fermentation and Aerobic Stability of Corn Silage. J. Dairy Sci..

[25] Buxton D.R., O’Kiely P.O., Buxton R.D., Muck R.E., Harrison J.H. (2003). Preharvest Plant Factors Affecting Ensiling. Silage Science and Technology.

[26] Gordon C.H., Derbyshire J.C., Wiseman H.G., Kane E.A., Melin C.G. (1961). Preservation and Feeding Value of Alfalfa Stored as Hay, Haylage, and Direct-Cut Silage. J. Dairy Sci..

[27] Gordon C.H., Kane E.A., Derbyshire J.C., Jacobson W.C., Melin C.G., McCalmont J.R. (1959). Nutrient Losses, Quality, and Feeding Values of Wilted and Direct-cut Orchardgrass Stored in Bunker and Tower Silos. J. Dairy Sci..

[28] Cheng Q., Chen Y., Bai S., Chen L., You M., Zhang K., Li P., Chen C. (2021). Study on the Bacterial Community Structure and Fermentation Characteristics of Fresh and Ensiled Paper Mulberry. Anim. Sci. J..

[29] Cai Y., Fujita Y., Murai M., Ogawa M., Yoshida N., Kitamura A., Miura T. (2003). Application of Lactic Acid Bacteria (*Lactobacillus Plantarum* Chikuso-1) for Silage Preparation of Forage Paddy Rice. J. Jap. Soc. Grassl. Sci..

[30] Wang S., Zhao J., Dong Z., Li J., Kaka N.A., Shao T. (2020). Sequencing and Microbiota Transplantation to Determine the Role of Microbiota on the Fermentation Type of Oat Silage. Bioresour. Technol..

[31] Singh V.K., Shukla K.S., Singh A.K., Choudhary K.K., Kumar A., Singh A.K. (2019). Impact of Climate Change on Plant–Microbe Interactions under Agroecosystems. Climate Change and Agricultural Ecosystems.

[32] Queiroz O.C.M., Ogunade I.M., Weinberg Z., Adesogan A.T. (2018). Silage review: Foodborne pathogens in silage and their mitigation by silage additives. J. Dairy Sci..

[33] Gomes A.L.M., Jacovaci F.A., Bolson D.C., Nussio L.G., Jobim C.C., Daniel J.L.P. (2018). Effects of Light Wilting and Heterolactic Inoculant on the Formation of Volatile Organic Compounds, Fermentative Losses and Aerobic Stability of Oat Silage. Anim. Feed Sci. Technol..

[34] Kleinschmit D.H., Kung L. (2006). A Meta-Analysis of the Effects of Lactobacillus Buchneri on the Fermentation and Aerobic Stability of Corn and Grass and Small-Grain Silages. J. Dairy Sci..

[35] Ning Y., Yan A., Yang K., Wang Z., Li X., Jia Y. (2017). Antibacterial Activity of Phenyllactic Acid against *Listeria Monocytogenes* and *Escherichia Coli* by Dual Mechanisms. Food Chem..

[36] Kung J.L., Sheperd A.C., Smagala A.M., Endres K.M., Bessett C.A., Ranjit N.K., Glancey J.L. (1998). The Effect of Propionic Acid-Based Preservatives on the Fermentation and Aerobic Stability of Corn Silage and a Total Mixed Ration. J. Dairy Sci..

[37] Kawaguchi H., Teramura H., Uematsu K., Hara K.Y., Hasunuma T., Hirano K., Sazuka T., Kitano H., Tsuge Y., Kahar P. (2015). Phenyllactic Acid Production by Simultaneous Saccharification and Fermentation of Pretreated Sorghum Bagasse. Bioresour. Technol..

[38] Prema P., Smila D., Palavesam A., Immanuel G. (2010). Production and Characterization of an Antifungal Compound (3-Phenyllactic Acid) Produced by *Lactobacillus plantarum* Strain. Food Bioprocess Technol..

[39] Xin H., Zhou J. (2018). Heterologous Expression of a Novel D-lactate Dehydrogenase from *Lactobacillus* Sp. ZX1 and its Application in D-phenyllactic Acid Production. J. Int. Biol. Macromol..

[40] Jones D.J.C. (1991). The Biochemistry of Silage. J. Agric. Sci..

[41] Parvin S., Wang C., Li Y., Nishino N. (2010). Effects of Inoculation with Lactic Acid Bacteria on the Bacterial Communities of Italian Ryegrass, Whole Crop Maize, Guinea Grass and Rhodes Grass Silages. Anim. Feed Sci. Technol..

[42] Xu D., Ding W., Ke W., Li F., Zhang P., Guo X. (2019). Modulation of Metabolome and Bacterial Community in Whole Crop Corn Silage by Inoculating Homofermentative Lactobacillus plantarum and Heterofermentative Lactobacillus Buchneri. Front. Microbiol..

[43] Ni K., Wang F., Zhu B., Yang J., Zhou G., Pan Y., Tao Y., Zhong J. (2017). Effects of Lactic Acid Bacteria and Molasses Additives on the Microbial Community and Fermentation Quality of Soybean Silage. Bioresour. Technol..

[44] Pang H., Qin G., Tan Z., Li Z., Wang Y., Cai Y. (2011). Natural Populations of Lactic Acid Bacteria Associated with Silage Fermentation as Determined by Phenotype, 16S Ribosomal RNA and recA Gene Analysis. Syst. Appl. Microbiol..

[45] Chun B.H., Lee S.H., Jeon H.H., Kim D.-W., Jeon C.O. (2017). Complete Genome Sequence of *Leuconostoc Suionicum* DSM 20241T Provides Insights into its Functional and Metabolic Features. Stand. Genom. Sci..

[46] Liu B., Huan H., Gu H., Xu N., Shen Q., Ding C. (2019). Dynamics of a Microbial Community during Ensiling and upon Aerobic Exposure in Lactic Acid Bacteria Inoculation-Treated and Untreated Barley Silages. Bioresour. Technol..

[47] Ogunade I.M., Jiang Y., Cervantes A.A.P., Kim D.H., de Oliveira A.S., Vyas D., Weinberg Z.G., Jeong K.C., Adesogan A.T. (2018). Bacterial Diversity and Composition of Alfalfa Silage as Analyzed by Illumina MiSeq Sequencing: Effects of *Escherichia Coli* O157:H7 and Silage Additives. J. Dairy Sci..

[48] Li L., Yuan Z., Sun Y., Kong X., Dong P., Zhang J. (2017). A Reused Method for Molasses-Processed Wastewater: Effect on Silage Quality and Anaerobic Digestion Performance of *Pennisetum*. Bioresour. Technol..

